# Statistical Analysis of Main and Interaction Effects on Cu(II) and Cr(VI) Decontamination by Nitrogen–Doped Magnetic Graphene Oxide

**DOI:** 10.1038/srep34378

**Published:** 2016-10-03

**Authors:** Xinjiang Hu, Hui Wang, Yunguo Liu

**Affiliations:** 1 College of Environmental Science and Engineering, Central South University of Forestry and Technology, Changsha 410004, P. R. China; 2 College of Natural Resources and Environment, South China Agricultural University, Guangzhou 510642, P. R. China; 3 College of Environmental Science and Engineering, Hunan University, Changsha 410082, P. R. China; 4 Key Laboratory of Environmental Biology and Pollution Control (Hunan University), Ministry of Education, Changsha 410082, P. R. China; 5 Institute of Bast Fiber Crops, Chinese Academy of Agricultural Sciences, Changsha, 410205, P. R. China

## Abstract

A nitrogen–doped magnetic graphene oxide (NMGO) was synthesized and applied as an adsorbent to remove Cu(II) and Cr(VI) ions from aqueous solutions. The individual and combined effects of various factors (A: pH, B: temperature, C: initial concentration of metal ions, D: CaCl_2_, and E: humic acid [HA]) on the adsorption were analyzed by a 2^5−1^ fractional factorial design (FFD). The results from this study indicated that the NMGO had higher adsorption capacities for Cu(II) ions than for Cr(VI) ions under most conditions, and the five selected variables affected the two adsorption processes to different extents. A, AC, and C were the very important factors and interactions for Cu(II) adsorption. For Cr(VI) adsorption, A, B, C, AB, and BC were found to be very important influencing variables. The solution pH (A) was the most important influencing factor for removal of both the ions. The main effects of A–E on the removal of Cu(II) were positive. For Cr(VI) adsorption, the main effects of A and D were negative, while B, C, and E were observed to have positive effects. The maximum adsorption capacities for Cu(II) and Cr(VI) ions over NMGO were 146.365 and 72.978 mg/g, respectively, under optimal process conditions.

Graphene is an attractive two-dimensional (2D) carbon material with a honeycomb structure and a thickness of one atom, where the carbon atoms are sp^2^-hybridized^1^,^2^,^3^. Graphene oxide (GO) is a precursor for graphene synthesis by chemical or thermal reduction processes, and is a specific branch of graphene research^4^. GO has a wide range of functional groups, such as hydroxyl, epoxide, carbonyl, and carboxyl, which makes it strongly hydrophilic, allowing it to readily swell and disperse in water^5^. In recent years, GO has emerged as a promising material for the removal of metal ions^6^,^7^,^8^ and organic contaminants^3^,^9^,^10^ from aqueous solutions. However, it is difficult to remove the suspended GO from the water after the adsorption process due to its high hydrophilicity, which possibly limits the direct application of GO-based materials in waste water treatment. The functional groups and large specific surface area of GO provide an excellent platform for loading magnetic nanoparticles^11^. The integration of magnetic properties into GO can combine the advantages of high adsorption capability with the merit of easy separation^8^,^11^,^12^.

The adsorption behavior of GO can be altered by changing the surface properties of the GO sheets by chemical functionalization. Recently, efforts have been made to synthesize chemically modified GO sheets by grafting organic moieties onto GO^13^. Examples include the grafting of polyaniline^14^, EDTA^8^,^15^, and β-cyclodextrin^16^ onto GO to improve its adsorption ability and selectivity for metal ions. As an amine derivative, diethylenetriamine (DETA) has two terminal amine groups and one central imine group, all of which are good donor groups for the formation of stable complexes with various metal ions^17^,^18^. Therefore, the introduction of DETA in a GO-based material would combine the unique properties of GO (large surface area and large number of functional groups) and the DETA (strong complexation ability to metal ions)^19^, providing good opportunities for application in the field of waste water treatment and metal ions recovery. We have previously reported a method to synthesize a magnetic graphene oxide composite (MGO) by coprecipitating Fe^2+^ and Fe^3+^ with ammonia solution in a GO solution^20^,^21^. This composite could be easily recovered by magnetic separation from the medium. In this study, we fabricated a GO-based material by grafting DETA to MGO (nitrogen-doped magnetic graphene oxide (NMGO)), and applied it as an adsorbent to remove metal ions from aqueous solution.

It is well known that the adsorption properties of a material for metal ions are affected by a number of factors, such as pH, temperature, concentration of the adsorbate, background electrolytes, and coexisting organic compounds^22^. In traditional experimental design, the effect of one factor is assessed by varying its value, and keeping all other factors constant^23^. This method can only study one factor at a time and cannot capture the interactions between factors^24^. The fractional factorial design (FFD) provides an option to weigh and quantify the relative importance of the examined factors and to assess possible interactions between them with a minimum number of experiments^25^,^26^. Moreover, it has the advantages of not requiring complicated calculations for analyzing the obtained data^27^,^28^,^29^.

Hence, the objectives of the current study were to (1) synthesize NMGO and characterize by high resolution transmission electron microscope (HRTEM), scanning TEM (STEM), Raman, x-ray diffraction (XRD), and x-ray photoelectron spectroscopy (XPS), (2) investigate the effects of pH, temperature, initial concentration of metal ions, CaCl_2_, and humic acid (HA) on the removal of Cu(II) and Cr(VI) ions from aqueous solution, (3) identify important factors and their interactions, and to optimize conditions for the adsorption of Cu(II) and Cr(VI) ions over NMGO by FFD.

## Results and Discussion

### Characterization

**Figure 1:**
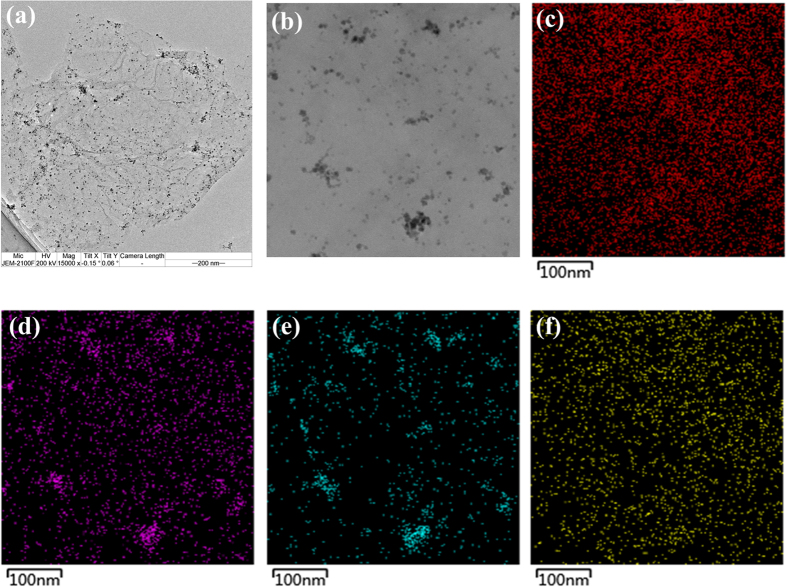
(**a**) HRTEM of NMGO; (**b**) STEM mapping of NMGO; (**c**) C element; (**d**) O element; (**e**) Fe element; (**f**) N element.

The morphology of the as-prepared NMGO composite was investigated by HRTEM (Fig. 1a), which revealed typical fabric-like shape and crumpled nanostructure of the thin and large sheet of GO^30^. Several small black spots (Fe_3_O_4_ nanoparticles) were dispersed on the planes of the GO. STEM was performed to identify the specific components of the NMGO (Fig. 1b–f). The presence of C from the graphitic 2D hexagonal lattice and the grafted DETA was visualized (Fig. 1c). Oxygen atoms (Fig. 1d), mainly from the oxygen-containing groups and the Fe_3_O_4_ nanoparticles, and Fe atoms (Fig. 1e) from the Fe_3_O_4_ nanoparticles, were distributed on the composite surface. The N atoms were assigned to the grafted DETA, and distributed evenly on the composite surface (Fig. 1f), demonstrating the successful grafting of DETA onto the MGO.

**Figure 2:**
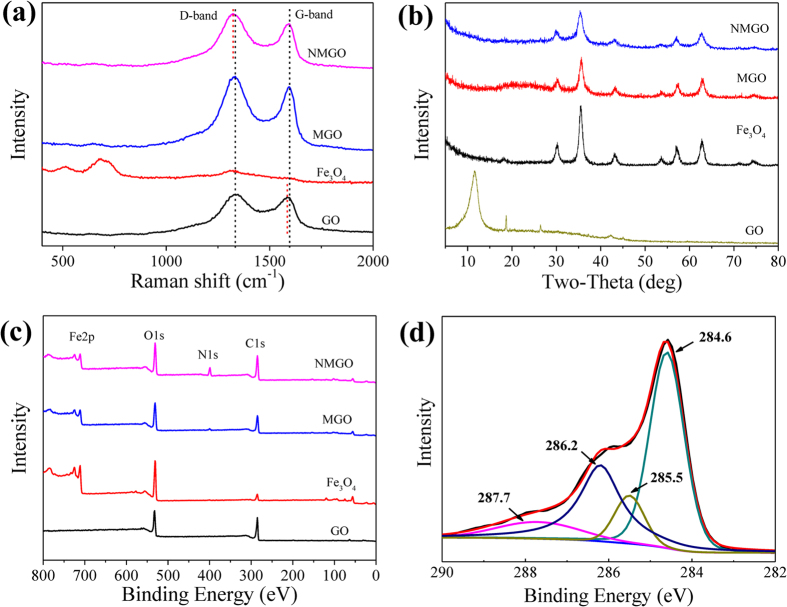
(**a**) Raman, (**b**) XRD, and (**c**) XPS survey scan spectra of GO, Fe_3_O_4_, MGO, and NMGO; (**d**) C1s XPS spectrum of NMGO.

The Raman spectra of GO, Fe_3_O_4_, MGO, and NMGO (Fig. 2a) show two prominent peaks around 1330 cm^−1^ and 1590 cm^−1^ (GO, 1334 and 1584 cm^−1^; MGO, 1334 and 1594 cm^−1^; NMGO, 1323 and 1594 cm^−1^), which were assigned to the D band and G band, respectively. Compared to GO and MGO, the D band in NMGO shifted to 1323 cm^−1^, which could be attributed to the introduction of DETA to the *sp*^2^ carbon network of MGO. The blue shift of the G-band in MGO and NMGO when compared to GO, indicated a strong electronic interaction between the GO and Fe_3_O_4_ nanoparticles^31^,^32^. Furthermore, the intensity ratios of the D (*I*_D_) and G bands (*I*_G_) of MGO (1.13) and NMGO (1.18) were slightly higher than that of GO (1.06), which could be due to the structural distortions induced by the anchored Fe_3_O_4_ nanoparticles and the grafted DETA^13^,^32^.

The XRD patterns of GO, Fe_3_O_4_, MGO, and NMGO are presented in Fig. 2b. GO shows a distinctive peak at 2θ = 11.58, due to the structure expansion caused by the incorporation of oxygen-containing groups between the carbon sheets during the course of strong oxidation^33^. In the XRD patterns of Fe_3_O_4_, MGO, and NMGO, the seven major peaks of the cubic spinel Fe_3_O_4_ (JCPDS Card No. 19-0629) were seen^20^,^34^, confirming that the synthesized MGO and NMGO composites contained a significant amount of Fe_3_O_4_ nanoparticles. In addition, the XRD patterns of Fe_3_O_4_, MGO, and NMGO were very similar, as the process of synthesis did not change the crystal structure of Fe_3_O_4_ microspheres^35^ and the weak carbon peaks of GO (in MGO and NMGO) were overwhelmed by the strong signals of the iron oxides^33^.

The chemical state of the elements in the samples was investigated by XPS. Figure 2c provides the survey spectra of GO, Fe_3_O_4_, MGO, and NMGO from 0–800 eV. For NMGO, photoelectron lines observed at about 285, 400, 531, and 710 eV were attributed to the binding energies of C1s, N1s, O1s, and Fe2p, respectively^33^. The XPS survey of NMGO shows a significant amount of N1s comparing to that of MGO, which originated from the grafted DETA. The C1s XPS spectrum of the NMGO obtained in high resolution is demonstrated in Fig. 2d. As reported by us previously^16^, the C1s XPS spectrum of MGO could be curve-fitted into five different peaks at 284.6, 286.2, 286.9, 288.1, and 289.0 eV, corresponding to C–C, C–O, C–O–C, C=O, and O–C=O groups, respectively. After reaction with DETA, the peaks at 286.9, 288.1, and 289.0 eV were absent in the C1s XPS spectrum of the NMGO, which might be due to the reaction of the carboxyl, carbonyl, and epoxide groups of MGO with DETA^16^. The appearance of the C–N peak at 285.5 eV and the HNC=O peak at 287.7 eV confirmed that the surface of MGO was functionalized with DETA^36^,^37^.

### Factors affecting the adsorbed amount of Cu(II) and Cr(VI)

**Figure 3:**
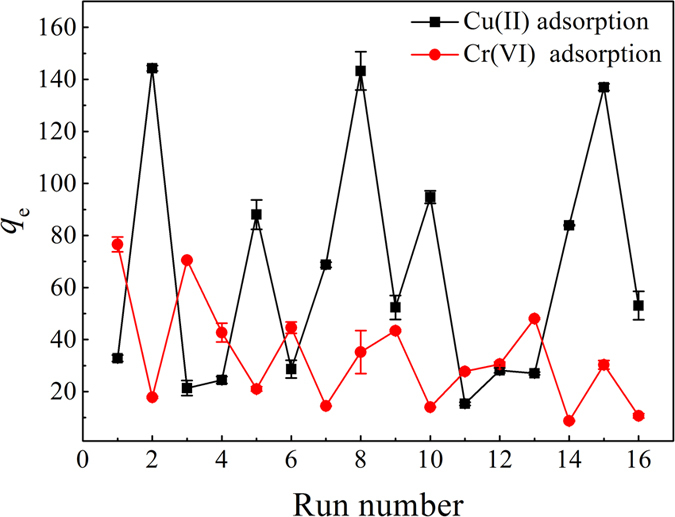
Experimental data obtained from the FFD experiments.

The effects of varying five factors (pH, temperature, initial concentration of metal ions, CaCl_2_, and HA) on the adsorption characteristics of NMGO for Cu(II) and Cr(VI) ions were studied via a 2^5−1^ FFD, and the results are demonstrated in Fig. 3. The adsorption capacities in the system with low (−) and high (+) levels of different factors were found to range from 15.33−144.27 mg/g for Cu(II) ions and from 8.73−76.56 mg/g for Cr(VI) ions, respectively, indicating the significance of the chosen parameters. The highest Cu(II) removal was observed in runs 2, 8, and 15 with adsorption capacities of 144.27, 143.23, and 136.98 mg/g, respectively. All runs had the same values of pH 6 and initial concentration of Cu(II) (50 mg/L), indicating that increasing the solution pH and copper concentration could enhance the Cu(II) adsorption. In contrast, the lower Cu(II) adsorption was found in runs 3, 4, and 11, where the pH and HA concentrations were low. From the Cr(VI) adsorption curve, runs 1 and 3 (with low pH, high temperature, and high Cr(VI) concentration) showed high Cr(VI) removal, while low Cr(VI) adsorption occurred in runs 14 and 16.

**Figure 4:**
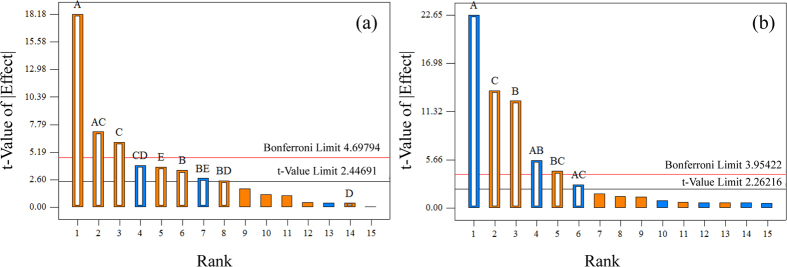
Pareto chart representing the effect of each factor on the adsorption of (**a**) Cu(II) and (**b**) Cr(VI) in the descending order from greatest to lowest contribution for FFD: (A) pH, (B) temperature, (C) initial concentration of metal ions, (D) CaCl_2_, (E) HA.

In order to study the significance of the FFD model in detail, the main effects of the factors and their interaction terms were evaluated by testing the probability (appropriate probability plots)^29^. The insignificant effects are normally distributed with mean zero and tend to fall along a straight line in the plot, while the significant effects show up as outliers on the normal probability plot^23^. Supplementary Fig. S8 shows the normal probability plot of the effects of factors and their interactions on Cu(II) and Cr(VI) removal. For Cu(II) adsorption (Fig. S8a), the significant effects were the main effects of A (pH), B (temperature), C ([Cu(II)]), and E ([HA]), and the interactions of AC (pH × [Cu(II)]), BD (temperature × [CaCl_2_]), BE (temperature × [HA]), and CD ([Cu(II)] × [CaCl_2_]). Although the main effect of CaCl_2_ concentration (D) was not a significant term for Cu(II) adsorption efficiency, to achieve hierarchic models, it was also included^38^. As seen in Fig. S8b, the factors that had significant effects on the Cr(VI) removal were A, B, and C, and their interactions of AB, AC, and BC. These results were verified by the Pareto chart (Fig. 4), where the Bonferroni limit is the threshold above which the effects that emerge are significant (very important). The effect terms below the threshold of the t-limit are insignificant factors. Effects emerging above the t-limit but below Bonferroni limit may possibly be significant (moderately important)^39^. Thus, for the adsorption of Cu(II) ions by NGMO (Fig. 4a), the factorial effects of very important main factors and their interactions were found to be in the following order: A > AC > C. The main factors of B and E and the interactions of BE, BD, and CD could be considered as moderately important. For the Cr(VI) adsorption (Fig. 4b), the very important influence variables were A, B, C, AB, and BC. AC was a moderately important interaction term.

Analysis of the variance (ANOVA) method was used for estimating the effects of factors and their interactions on Cu(II) (Supplementary Table S2) and Cr(VI) (Supplementary Table S3) removal (response). From the tables, the F-value of 52.66 for Cu(II) removal and 152.95 for Cr(VI) removal indicated that the models were statistically significant at the designated conditions^38^. Prob > F-values less than 0.05 indicate model terms are significant. In the system for Cu(II) removal, A, B, C, E, AC, BD, BE, and CD were the significant model terms. A, B, C, AB, AC, and BC were the main significant factors for the removal of Cr(VI). The values of the predicted *R*^2^ and adjusted *R*^2^ were calculated by the present fractional factorial model, and were found to be in reasonable agreement for both Cu(II) (9875 and 0.9687, respectively) and Cr(VI) adsorption (0.9903 and 0.9838, respectively). Adequate precision measures the signal to noise ratio, where a ratio greater than 4 is desirable. The ratios obtained in this study were 20.821 and 37.025, indicating an adequate signal. The resulting models for predicting the Cu(II) and Cr(VI) adsorption can be represented by the following equations:

For Cu(II) adsorption in coded factors:


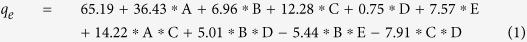


in actual factors:


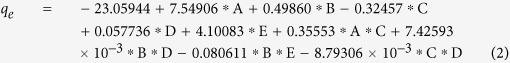


For Cr(VI) adsorption in coded factors:


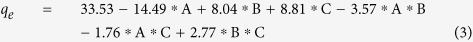


in actual factors:


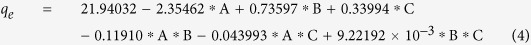


**Figure 5:**
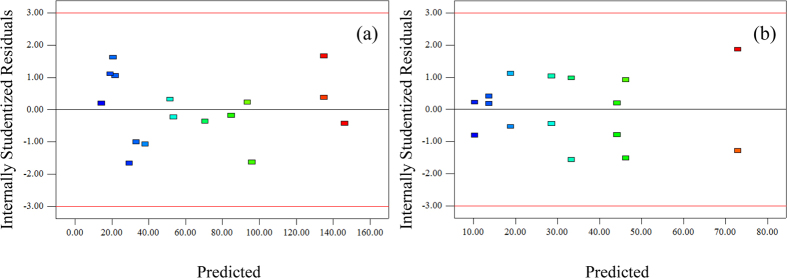
Plots of internally standardized residuals with predicted values for (**a**) Cu(II) and (**b**) Cr(VI) adsorption.

A normal probability plot of studentized residuals and a plot of the residuals versus predicted response values can be used to test the validity of the above selected model^29^. From the normal probability plot of studentized residuals (Supplementary Fig. S9), all internally studentized residuals lie close to the straight lines, which means that a normal pattern was observed for the regression residuals^39^. Supplementary Fig. S10 shows the predicted data versus actual values for Cu(II) and Cr(VI) adsorption onto NMGO. These plots represent the predictive ability of the models over a range of data, and the plots should exhibit a random scatter around a 45° line^38^. According to Fig. S10, all the values predicted by the selected model were very close to the experimental measurements, indicating that the Cu(II) and Cr(VI) adsorption onto NMGO could be predicted by the obtained FFD models. The adequacy of the obtained models was also evaluated by the plot of studentized residuals versus predicted values of the responses (Fig. 5). The internally studentized residuals were equally scattered above and below the x-axis in the range of −3 and +3, indicating that the proposed model was adequate and there is no reason to suspect any violation^29^,^38^,^40^.

### Analysis of significant main examined factors and their interactions

**Figure 6:**
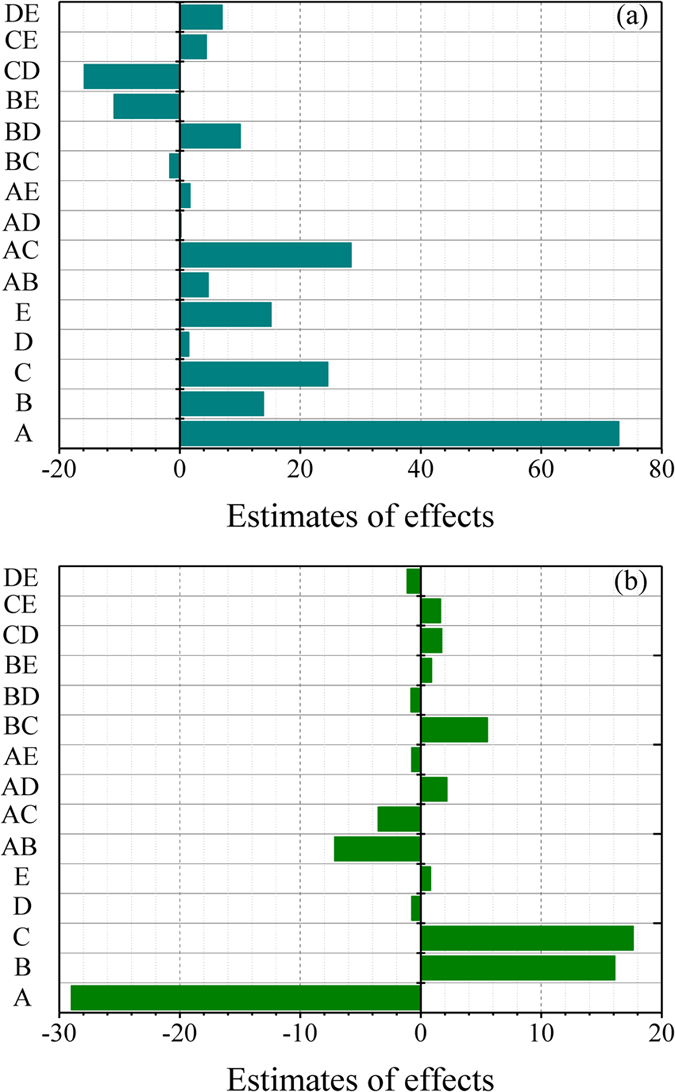
Identification of main effective factors and interaction factors on (**a**) Cu(II) and (**b**) Cr(VI) removal by NMGO: (A) pH, (B) temperature, (C) initial concentration of metal ions, (D) CaCl_2_, (E) HA.

The estimates of 2^5−1^ FFD are showed in Fig. 6. The main effect for each of the variables is the difference between the average responses of high level variables (+) and low level variables (−) in the design matrix of Supplementary Table S4^28^,^41^. From Fig. 6a, the positive estimates of the main effects of A–E indicated that adsorption capacity of NMGO for Cu(II) ions increased when the variables increased from low level to high level. As seen from Fig. 6b, the negative estimates of main effects of A and D were −28.99 and −0.75, respectively, while positive effects were observed for B (16.09), C (17.62), and E (0.79). That is to say, increasing the solution pH from 2 to 6 could significantly increase the Cu(II) adsorption, while decrease the Cr(VI) adsorption. It is well known that the solution pH not only affects the speciation of Cr(VI), but also the surface charge of the sorbents^16^. The pH_pzc_ value (point of zero charge) for NMGO was measured to be 5.94. At pH < 5.94, the zeta potentials were positive, whereas the NMGO surfaces were negatively charged at pH > 5.94. As the pH increased, the electrostatic repulsion between Cu(II) ions (Cu^2+^) and the sorbent reduced, which increased the Cu(II) sorption. For Cr(VI), the negatively charged HCrO_4_^−^ was easily attracted by the positively charged surfaces of NMGO in low pH value environment. With increasing pH, the decrease of zeta potentials resulted in a decrease in electrostatic attraction between HCrO_4_^−^ and the NMGO, leading to a decrease in Cr(VI) adsorption.

**Figure 7:**
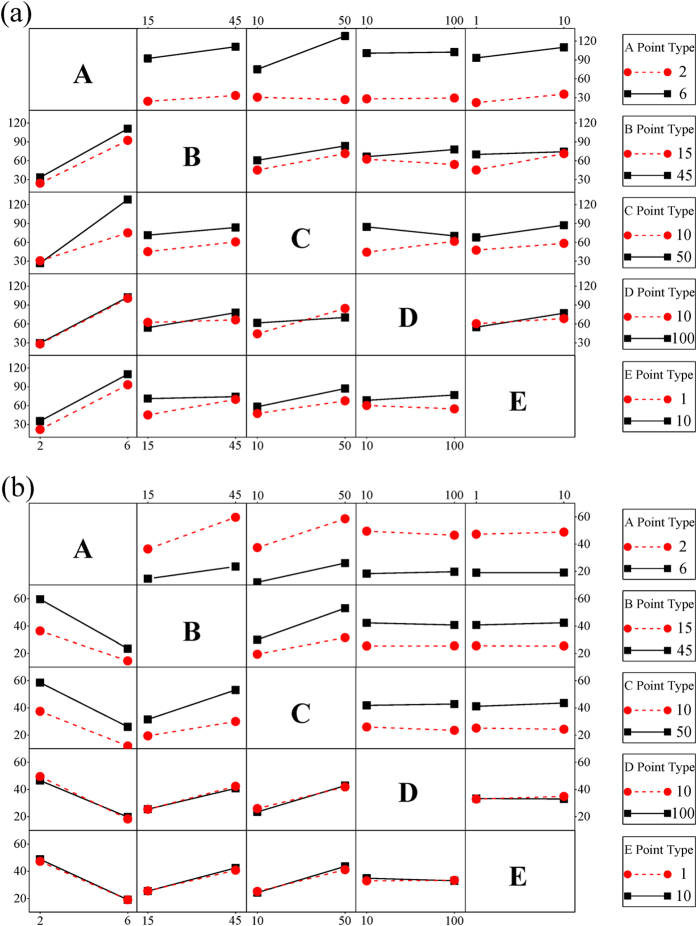
Interaction effects plot for (**a**) Cu(II) and (**b**) Cr(VI) removal: (A) pH, (B) temperature, (C) initial concentration of metal ions, (D) CaCl_2_, (E) HA.

Generally, the factors investigated in this study simultaneously affect the adsorption processes in practical engineering. Therefore, the mutual effects of any two factors should be assessed. To this aim, the interaction plots (Fig. 7) allowed one to analyze in depth the possible combined effects of the factors considered in the FFD. If the two lines in the cell of the plot are parallel, the two factors have no interactions. Conversely, non-parallel lines indicated an interaction between the two factors^42^,^43^, and a larger angle between two lines in the cell indicated a stronger interaction^44^. The interaction effects of the five factors for Cu(II) removal (Fig. 7a) show that the lines in cells AD (or DA), AE (or EA), and BC (or CB) were nearly parallel, suggesting that the two variables in these cells had few interactions. The angle between two lines in cell AC (or CA) was larger than others, indicating that the solution pH (A) and Cu(II) concentration (C) could affect each other significantly. We could also single out important factors for interaction within each cell^41^. In columns A and C (Fig. 7a), all adsorption capacities of NMGO for Cu(II) in cells, except the cell AC (low A), increased with increasing solution pH and Cu(II) concentration from low levels to high levels. These results are consistent with the findings in Pareto chart (Figs 4 and 5. Supplementary Fig. S11 shows the 2D contour curves and 3D surface response plot for AC interaction in the adsorption of Cu(II). The simultaneous increase of both factor (AC) resulted in the improvement of Cu(II) removal by the NMGO. At lower levels of pH, the change of initial Cu(II) concentration proved to exert a small impact on Cu(II) adsorption. We can also see that a high pH was very important for the adsorption process, and this influence was particularly promoted by higher initial Cu(II) concentration.

The combined effects (Fig. 7b) of AB (or BA), BC (or CB), and AC (or CA) had strong interaction for Cr(VI) removal because the lines in these cells are converging. In column A, the Cr(VI) adsorption in the cells decreased with increasing pH values from low level (2) to high level (6). From the cells in columns B and C, when the temperature and Cr(VI) concentration increased from low to high level, the Cr(VI) removal increased slightly. The lines in columns D and E show near zero slopes, suggesting that factors D and E imposed slight or no impact on Cr(VI) decontamination. 2D contour curves and the 3D surface response plot for AB, BC, and AC interactions in the Cr(VI) adsorption are showed in Supplementary Fig. S12. According to Fig. S12a,b, the adsorption capacities of NMGO decreased when pH increased from 2 to 6, but it increased as the temperature increased from 15 to 45 °C. From Fig. S12c,d, adsorption also improved upon the increase of the Cr(VI) concentration. Figure S12e,f revealed that the increase of the temperature and the Cr(VI) concentration led to an increase in the amount of adsorbed Cr(VI).

### Optimization and comparison of influencing factors on adsorption of Cu(II) and Cr(VI)

To determine the optimum levels for the significant factors, the ultimate optimum conditions were assessed. The goal of optimizing operational conditions is to achieve a balance between the high adsorption efficiency toward metal ions and low process expenses^29^. The five factors were set within the range given in Table 1, and the maximum adsorbed amounts of metal ions were chosen as the optimizing target. The maximum adsorption capacity (*q*_m_) of NMGO for Cu(II) ions (146.365 mg/g) was achieved by a combination of the following parameters: pH = 6, temperature = 15 °C, initial concentration of copper = 50 mg/L, CaCl_2_ concentration = 10 mmol/L, and HA concentration = 10 mg/L. The *q*_m_ of NMGO for Cu(II) is larger than those of GO (117.5 mg/g at room temperature, 1 g/L dosage, pH 5.3)^45^, GO/Fe_3_O_4_ (18.26 mg/g at 20 °C, 0.4 g/L dosage, pH 5.3)^11^, and GO aerogel (19.65 mg/g at 25 °C, 0.6 g/L dosage, pH 6.3)^46^. The optimal process conditions for Cr(VI) adsorption (72.978 mg/g) determined by FFD method were as follows: pH = 2, temperature = 45 °C, initial concentration of copper = 50 mg/L, CaCl_2_ concentration* = 100 mmol/L, and HA concentration* = 1 mg/L (The D and E terms were not included in the model). The *q*_m_ of NMGO for Cr(VI) is larger than those of EMCMCR (48.78 mg/g at 30 °C, 5 g/L dosage, pH 2.0)^47^, CD-E-MGO (68.41 mg/g at 30 °C, 0.17 g/L dosage, pH 3.0)^16^, CTAB-GN (21.59 mg/g at 40 °C, 4 g/L dosage, pH 2.0)^48^.

The results of FFD indicated the variables which are important in the removal of Cu(II) and Cr(VI) ions. The NMGO had higher adsorption capacities for Cu(II) ions than those for Cr(VI) ions under most conditions. For example, The largest adsorption amount was 144.27 mg/g for Cu(II) ions and 76.56 mg/g for Cr(VI) ions. The adsorption capacities for Cu(II) and Cr(VI) under optimal process conditions were 146.365 and 72.978 mg/g, respectively. These results indicated that the NMGO performed better for the removal of Cu(II) ions. This phenomenon could be explained by the different adsorption mechanism of NMGO for Cu(II) and Cr(VI) ions. In the pH range of 2–6, the main Cu(II) and Cr(VI) species were Cu^2+^ and HCrO_4_^−^, respectively^16^,^44^. The amino groups of the grafted DETA had higher affinity for Cu^2+^ than HCrO_4_^−^. Cu^2+^ adsorption occurred mainly by the formation of inner-sphere complexes, while HCrO_4_^−^ was most likely adsorbed as outer-sphere complexes^49^,^50^. The solution pH was the most important factor that influenced the removal of the two metal ions. An increase of pH value was found to improve Cu(II) adsorption while inhibiting Cr(VI) removal. The temperature had a higher influence on the adsorption of Cr(VI) than Cu(II). The concentration of CaCl_2_ in the solution had a slight effect on the Cr(VI) and Cu(II) removal. HA was a moderately important factor for Cu(II) uptake, while it has a lesser influence on Cr(VI) decontamination, which could be explained by the complexation of surface-adsorbed HA and Cu(II)^20^. Also, HA could be adsorbed on the surface of NMGO by means of a π–π electron coupling, and the carboxyl groups (–COOH) of the adsorbed HA could then act as adsorption sites for Cu(II) ions by means of electrostatic interaction and ion exchange^51^.

## Conclusions

The results of analysis by HRTEM, STEM, Raman, XRD, and XPS indicated that the NMGO was successfully fabricated by grafting DETA onto MGO. The NMGO could be easily separated by a magnet, and therefore was suitable for application as an adsorbent for removing Cu(II) and Cr(VI) from aqueous solution. Fractional factorial design (FFD) was an appropriate statistical technique to study the effects of multiple factors on the adsorption process. In the system with low (−) and high (+) levels of different factors, the adsorption capacities were found to range from 15.33–144.27 mg/g for Cu(II) and 8.73–76.56 mg/g for Cr(VI), respectively. The single factors of A and C, and their interaction (AC) were very important factors for adsorption of Cu(II) ions, and they were found to be in the following order: A > AC > C. B, E, BE, BD, and CD could be considered as moderately important factors. A, B, C, AB, and BC were the parameters that strongly affected Cr(VI) adsorption, and AC could be taken as moderately important interaction term. Thus, the adsorption of Cu(II) and Cr(VI) on NMGO could be accurately predicted by the FFD models. The main effects of A–E on Cu(II) adsorption were positive. For Cr(VI) removal, main effects of A and D were negative, while those of B, C, and E were positive. The influence of the solution pH on Cu(II) adsorption was particularly promoted by a higher initial Cu(II) concentration. The experimental set with the maximum desirability value yielded adsorbed amounts of 146.365 and 72.978 mg/g for Cu(II) and Cr(VI), respectively, under optimum conditions.

## Materials and Methods

### Synthesis of GO, Fe_3_O_4_, MGO, NMGO

**Figure 8:**
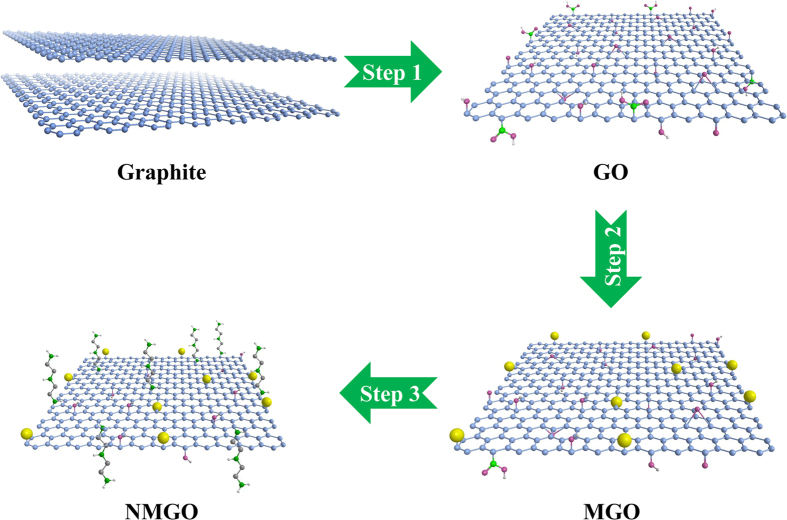
The proposed scheme for the formation of NMGO: (Step 1) preparation of graphene oxide; (Step 2) synthesis of MGO by loading magnetic nanoparticles on the GO surface; (Step 3) formation of NMGO by grafting DETA to the MGO surface.

Graphene oxide (GO) was prepared from natural graphite by the modified Hummers method^20^,^52^. The graphite powders were first preoxidized, and then treated with concentrated H_2_SO_4_, KMnO_4_, and NaNO_3_ in order to promote oxidation. The excess MnO_4_^−^ ions were eliminated by adding H_2_O_2_ solution. The obtained products were rinsed thoroughly with HCl solution and Milli-Q water. The obtained adhesive graphite oxide layers were separated from each other by ultrasonication and a GO solution was obtained. The magnetic graphene oxide (MGO) was prepared by the coprecipitation method^20^,^53^. Briefly, Fe^3+^ and Fe^2+^ were mixed in the GO solution, and then the ammonia solution was added into the mixture to form the Fe_3_O_4_@GO composite (MGO)^20^,^21^,^53^. For comparison, we simultaneously prepared pure Fe_3_O_4_ without GO. The nitrogen-doped magnetic graphene oxide (NMGO) composite was prepared by introducing DETA into the MGO composite^36^. For this, 500 mL of MGO suspension and 4.5 mL ammonia solution were mixed and the suspension was continually stirred at 15–30 °C for 5 min. Then, 18 mL of diethylenetriamine was added slowly and the mixture stirred for 10 min, followed by heating at 95 °C for 6 h. The obtained NMGO was washed repeatedly with ethanol and Milli-Q water and stored at room temperature. The synthesis of NMGO is schematically represented in Fig. 8.

### Characterization

The morphology of NMGO was characterized by HRTEM and STEM (JEM-2100F, Japan). The Raman spectra were carried out using a Raman spectrometer (Labram-010, JY, FAR). The XRD patterns were obtained on a Rigaku D/max-2500 diffractometer equipped with a rotating anode and Cu *K*α source. The XPS measurements were performed using an ESCALAB 250Xi X-ray photoelectron spectrometer (Thermo Fisher, USA). The zeta potentials were measured using a Zetasizer Nano SZ (ZEN3690, Malvern, UK).

### Fractional factorial design and sorption experiments

In this work, five factors, namely pH, temperature, initial concentration of metal ions, CaCl_2_, and HA, were screened for their effects on the adsorption capacities of NMGO for Cu(II) and Cr(VI) ions (response) using a 2^5−1^ FFD with resolution V. The experimental factors and their levels used in the 2^5−1^ FFD were demonstrated in Table 1. The software of Design Expert 8.0.6 (Stat-Ease Inc., Minneapolis, MN, USA) was used for the FFD of the experiments and regression analysis of the obtained experimental data. The full design matrix of the 2^5−1^ FFD is shown in Supplementary Table S4. All adsorption experiments were performed in a 150 mL conical flask containing 50 mL aqueous solution of Cu(II) or Cr(VI), and the samples were agitated at 150 rpm for 24 h in a rotary shaker. Then, the mixture in the conical flask was separated using a permanent magnet. The Cu(II) and Cr(VI) concentrations in the supernatant were analyzed by a flame atomic absorption spectrometry (PerkinElmer AA700, USA) and an UV-visible spectrophotometer (Pgeneral T6, Beijing) at 540 nm, respectively^16^. The adsorption capacity (*q*_e_) of NMGO was calculated from the difference between the initial concentration (*C*_0_) and the equilibrium concentration (*C*_e_).

### Statistical Analysis

All measurements in this study were conducted in triplicate. An analysis of variance was used to evaluate the significance of results, and p < 0.05 was considered to be statistically significant.

## Additional Information

**How to cite this article**: Hu, X. *et al*. Statistical Analysis of Main and Interaction Effects on Cu(II) and Cr(VI) Decontamination by Nitrogen–Doped Magnetic Graphene Oxide. *Sci. Rep*. **6**, 34378; doi: 10.1038/srep34378 (2016).

## Supplementary Material

Supplementary Information

## Figures and Tables

**Table 1:** Experimental factors.

Factors		(−)	0	(+)
A	pH	2	4	6
B	Temperature (°C)	15	30	45
C	Initial concentration of metal ions (mg/L)	10	30	50
D	CaCl_2_ (mmol/L)	10	55	100
E	HA (mg/L)	1	5.5	10
