# Supplementary material for: Statistical Analysis of Main and Interaction Effects on Cu(II) and Cr(VI) Decontamination by Nitrogen–Doped Magnetic Graphene Oxide
Source: Sci Rep. 2016 Oct 3;6:34378. doi: 10.1038/srep34378 (PMC5046064; doi:10.1038/srep34378)
Supplement: Supplementary Information [file srep34378-s1.pdf]

Supplementary Information

Statistical Analysis of Main and Interaction Effects on Cu(II) and Cr(VI) Decontamination by Nitrogen–Doped Magnetic Graphene Oxide

Xinjiang Hu[1,2,3,4], Hui Wang[3,4,5], Yunguo Liu[3,4]

[1] College of Environmental Science and Engineering, Central South University of Forestry and Technology, Changsha 410004, P.R. China.

[2] College of Natural Resources and Environment, South China Agricultural University, Guangzhou 510642, P.R. China.

[3] College of Environmental Science and Engineering, Hunan University, Changsha 410082, P.R. China.

[4] Key Laboratory of Environmental Biology and Pollution Control (Hunan University), Ministry of Education, Changsha 410082, P.R. China.

[5] Institute of Bast Fiber Crops, Chinese Academy of Agricultural Sciences, Changsha, 410205, P.R. China.

Correspondence and requests for materials should be addressed to X.H. (email: huxinjiang@126.com) or Y.L. (email: hnuese@126.com)

Characterization. Fourier-transform infrared spectroscopy (FT-IR) spectra were collected by using a spectrophotometer (Nicolet 6700, USA). TG curves were recorded using thermoanalytical equipment (STA 409 PC, NETZSCH, Germany). The magnetic property was characterized by recording the magnetization curve using a vibrating sample magnetometer (Lake Shore 7410, USA). The elemental analyses were carried out with an elemental analyzer (Vario EL III, Elementar, Germany).

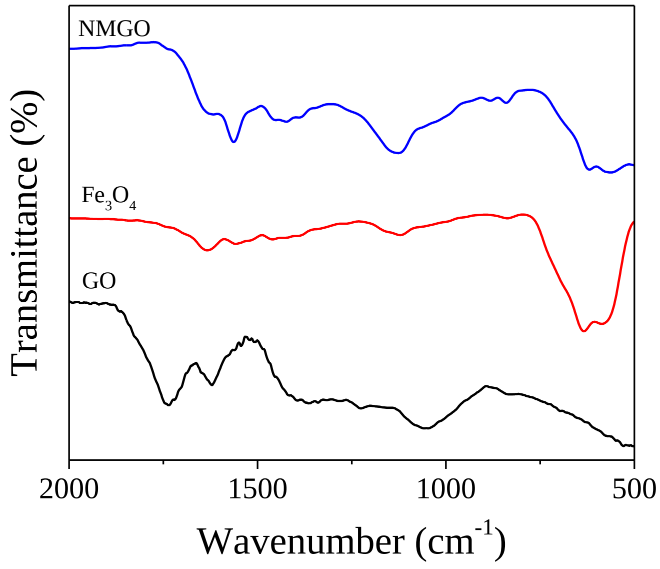

Figure S1. FTIR spectra of GO, Fe_3O_4, and NMGO.

The FT-IR spectra of GO, Fe_3O_4, and NMGO (Fig. S1) showed the characteristic bands[1-3] of various functional groups on the GO nanosheets, i.e. C=O at 1732 cm^{-1}, C=C at 1621 cm^{-1}, C–O at 1231 cm^{-1}, and C–O–C at 1050 cm^{-1}. In the spectrum of NMGO, the bands at 1564 and 1126 cm^{-1} correspond to the amide N−H bending mode and the stretching vibration of C–N[3], respectively, confirming the successful linking of DETA onto MGO. The characteristic band of the magnetic Fe_3O_4 nanoparticles at 620 cm^{-1}, attributed to the Fe–O bonds in the tetrahedral sites was also observed[4, 5].

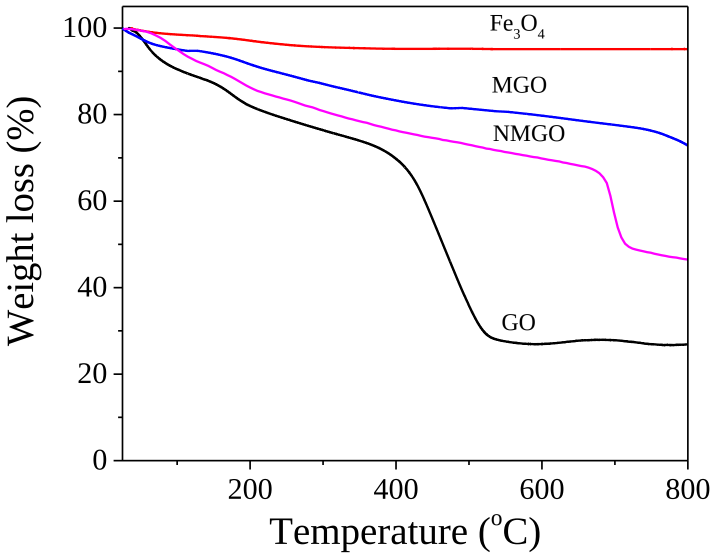

Figure S2. Thermogravimetric spectra of GO, Fe_3O_4, MGO, and NMGO.

Fig. S2 shows differential TG curves of GO, Fe_3O_4, MGO, and NMGO in the temperature range of 25–800 ℃. The first weight loss of GO occurs below 200 ℃ and was assigned to the residual water. The second and third weight loss around 200 and 400 ℃, are ascribed to the loss of CO and CO_2 from the decomposition of oxygen functional groups and carbon oxidation, respectively[2]. GO, Fe_3O_4, and MGO lose about 73.09 %, 4.88 %, and 26.08 %, respectively, of their total weight upon heating to 800 ℃. The NMGO was not thermally stable, and began to lose mass below 100 ℃. While the total mass loss of NMGO was 44.56 %, nearly 33.00% was lost between 25–660 ℃. The weight of the NMGO composite decreased rapidly as the temperature increased from 660 to 800 ℃, due to the continuous decomposition of the grafted DETA.

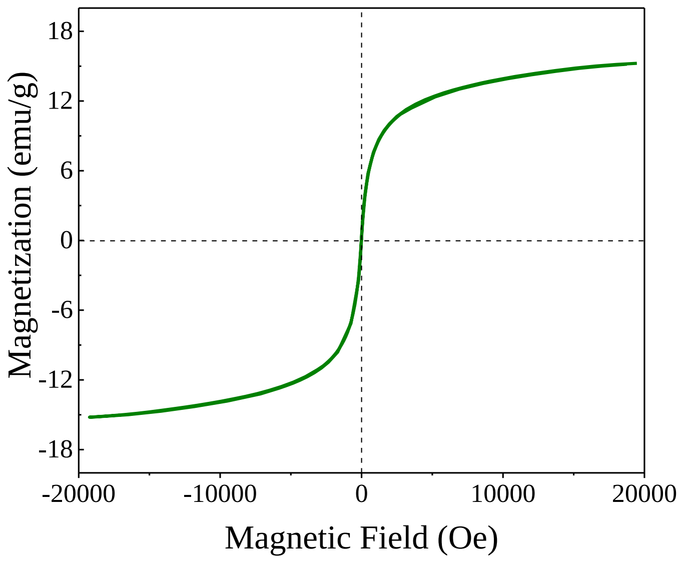

Figure S3. Magnetization curve of NMGO at room temperature.

The Fe_3O_4 nanoparticles on the surface of GO sheets endow NMGO with magnetic properties, and this is important for the convenient recycling of this adsorbent. When NMGO is added to the waste water, the pollutants are captured by the adsorption sites on the surface of the NMGO. The pollutant-loaded NMGO is then conveniently separated from the solution by a magnet. These pollutants on the NMGO can be desorbed and used as a resource, while the regenerated adsorbent can be reused. The magnetization spectrum of NMGO was measured by vibrating sample magnetometry (VSM) to estimate its negligible coercivity and remanence (Fig. S3). The saturation magnetization (M_s) was calculated to be 15.225 emu/g. The extremely small retentivity (0.059 emu/g) of the composite suggested that NMGO exhibited a superparamagnetic behavior at room temperature[6].

The elemental composition (C, H, and N) of NMGO was measured and the results are shown in Table S1. The percentages of C, H, and N in the NMGO were 30.71%, 2.14%, and 3.82%, respectively. The ratios of H/C and C/N were 0.070 and 8.039, respectively.

Elemental composition (%, mass based)				
C	H	N	H/C	C/N
30.710	2.14	3.82	0.07	8.039

Table S1. Elemental composition of NMGO.

Comparison experiments. The adsorption capacities of GO, MGO, and NMGO were compared at pH 6 for Cu(II) and pH 2 for Cr(VI), and the results are shown in Fig. S4. We can see that MGO has lower removal capacities for Cu(II) and Cr(VI) than GO. This could be because the Fe_3O_4 particles loaded on the GO surface might occupy some of the active sites of the GO. Also, the Fe_3O_4 particles might reduce the repulsion between GO nanosheets, leading to aggregation of GO, resulting in further loss of adsorption sites[7]. We can also see that the adsorption amounts of NMGO for Cu(II) and Cr(VI) were more than twice that of MGO, and higher than those of GO. These results showed that the DETA was successfully grafted onto the MGO surface, and has a positive effect on the adsorption process. DETA contains three amino groups, which can form stable complexes with various metals, increasing the adsorption capacity of MGO[8].

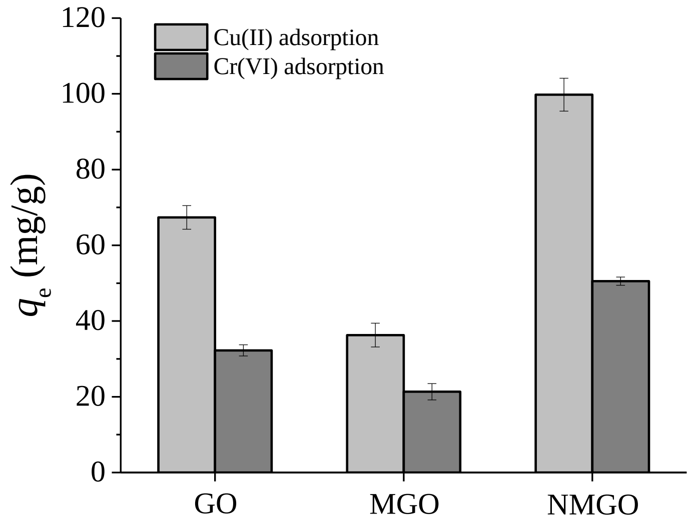

Figure S4. Comparison of adsorption capacities of GO, MGO, and NMGO for Cu(II) and Cr(VI) ions: pH = 6 (for Cu(II) adsorption), pH = 2 (for Cr(VI) adsorption), temperature = 30 ℃, initial concentration of copper or chromium = 30 mg/L, $CaCl_2$ concentration = 55 mmol/L and HA concentration = 5.5 mg/L, time = 24 h, adsorbent dosage = 96 mg/L.

Regeneration of used adsorbent. The recycling of NMGO in the adsorption of Cu(II) and Cr(VI) was investigated. Desorption of the adsorbed Cu(II) and Cr(VI) was carried out using 0.1 M EDTA and 0.1 M NaOH. The regenerated NMGO was reused for up to 5 cycles and the results are illustrated in Fig. S5. It was found that the adsorption capacities of NMGO for Cu(II) and Cr(VI) remained at 90 and 88% after 5 adsorption–desorption cycles, respectively. Thus, NMGO has a good reusability, and a potentially bright future for heavy metal removal from waste water.

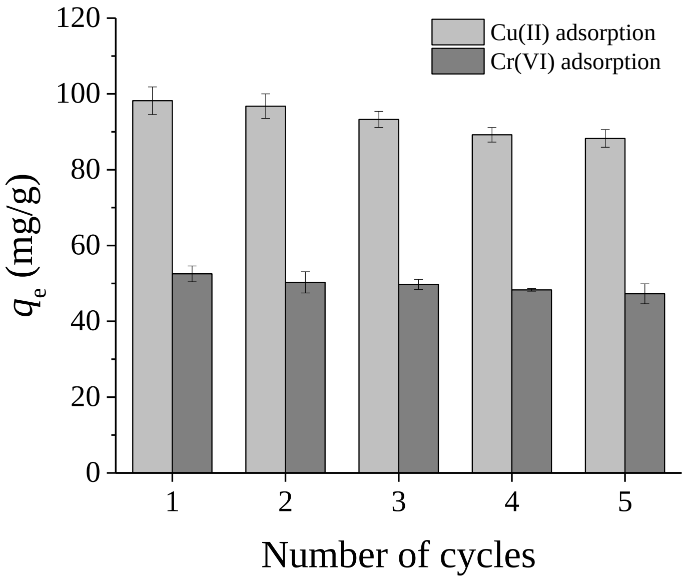

Figure S5. Reusability of the NMGO for Cu(II) and Cr(VI) removal (Adsorption conditions: pH = 6 (for Cu(II) adsorption), pH = 2 (for Cr(VI) adsorption), temperature = 30 ℃, initial concentration of copper or chromium = 30 mg/L, $CaCl_2$ concentration = 55 mmol/L and HA concentration = 5.5 mg/L, time = 24 h, adsorbent dosage = 96 mg/L; Desorption conditions: desorption solution, 0.1 M EDTA (for Cu(II) desorption) and 0.1 M NaOH (for Cr(VI) desorption), time = 2 h.).

Removal mechanism. In order to investigate the removal mechanism of Cu(II) and Cr(VI) over NMGO, FTIR and XPS analysis was carried out. The FTIR spectra of NMGO, before and after adsorption of Cu(II) and Cr(VI) are shown in Fig. S6. The FTIR spectrum of Cu(II)-adsorbed NMGO showed that the peaks expected at 1126 and 1564 cm^{-1} were shifted to 1120 and 1562 cm^{-1}, respectively, after adsorption. For Cr(VI) adsorption, the two peaks were shifted to 1128 and 1560 cm^{-1}, respectively. In addition, the peak intensity at ~1564 cm^{-1} decreased after the Cu(II) and Cr(VI) adsorption. These results indicated that the amino groups of the grafted DETA might be directly involved in the formation of surface complexes with Cu(II) and Cr(VI) ions[9, 10]. The XPS spectra of NMGO before and after adsorption of Cu(II) and Cr(VI) are shown in Fig. S7. The survey spectra after adsorption of Cu(II) and Cr(VI) ions showed peaks of Cu and Cr at ~934 and 576 eV, respectively, compared to the

survey spectra before adsorption (Fig. 2c). The N1s peak of NMGO was dissected into three peaks (Fig. S7b) at 398.4, 399.7, and 401.5 eV, which correspond to the nitrogen in the neutral imine (=N–), amine (–NH– or $–NH_2$), and protonated amine ($–NH_2^+–$ or $–NH_3^+$), respectively[10-13]. The N1s spectrum of NMGO after Cu(II) adsorption (Fig. S7c) shows that the peaks at 399.7 and 401.5 eV have shifted to 400.1 and 402.3 eV, respectively. Besides, a new peak at 406.7 eV was observed, which could be assigned to the nitrogen coordinated with Cu(II)[10]. From Fig.S7d, the binding energies of Cu2p at 932.6, 934.0, 942.6, and 953.1 eV revealed that the removal of Cu^{2+} was accompanied by the formation of Cu hydroxides[14]. In Fig. S7e, the binding energies of the N1s shifted to 400.1 and 402.1 eV after Cr(VI) adsorption, indicating that the amino groups participated in the adsorption. In the Cr2p spectrum after adsorption (Fig. S7f) the Cr2p peaks could be curve-fitted with three components at binding energies of 577.0, 579.3, and 586.8 eV, assigned to Cr(III) $2p_{3/2}$, Cr(VI) $2p_{3/2}$, and Cr(III) $2p_{1/2}$, respectively[9, 15]. These results suggested that both Cr(III) and Cr(VI) existed simultaneously on the surface of NMGO after adsorption. The Cr(III) on the NMGO surface originated mainly from chemical reduction interactions[12]. In an acidic environment, the Cr(VI) ions were captured by the amino groups of the NMGO, and then reduced to Cr(III) by the π-electrons on the carbocyclic six-membered ring of the GO and the photo electrons produced by the anchored Fe_3O_4 nanoparticles[3].

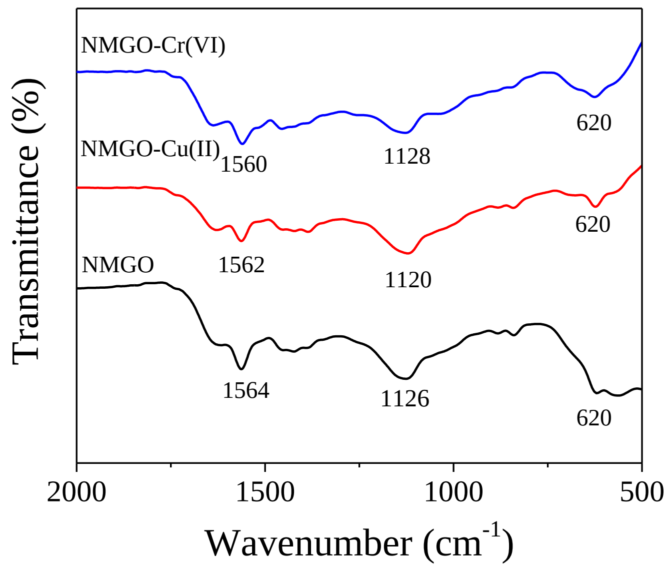

Figure S6. FTIR spectra of NMGO before and after adsorption of Cu(II) and Cr(VI).

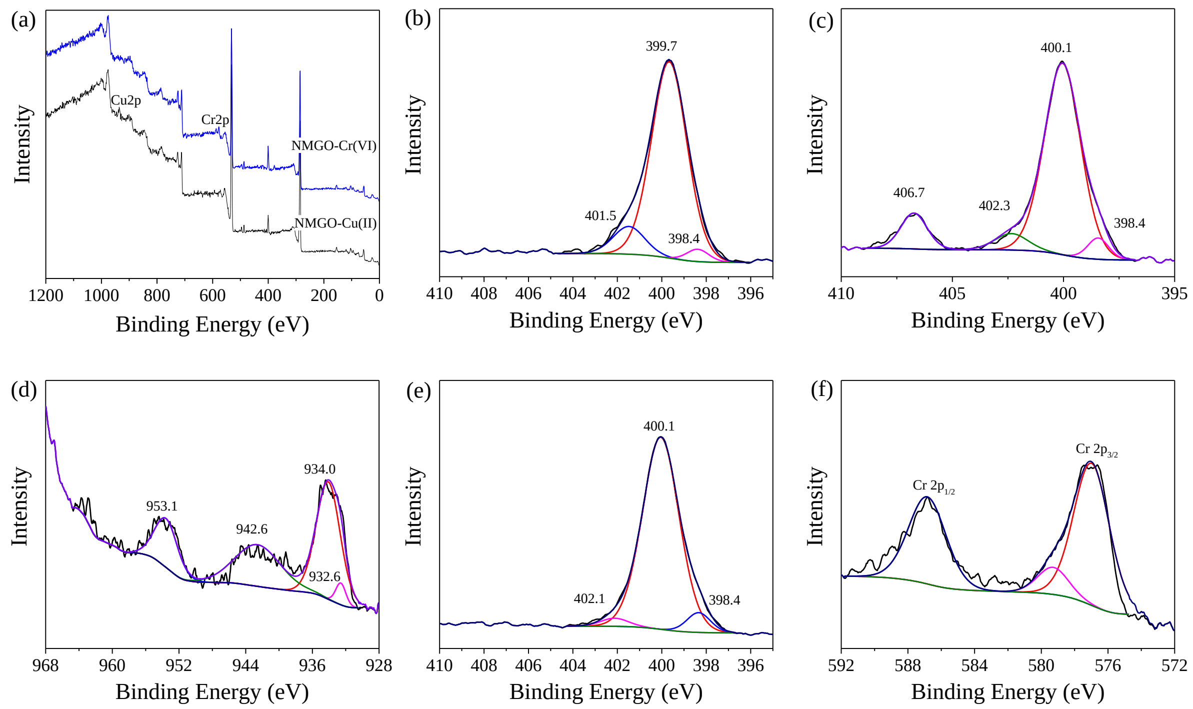

Figure S7. XPS spectra of NMGO: (a) XPS wide-scan of NMGO after adsorption of Cu(II) and Cr(VI); (b) N 1s before adsorption; (c) N 1s after adsorption of Cu(II); (d) Cu 2p high resolution XPS spectra of the Cu(II)-adsorbed NMGO; (e) N 1s after adsorption of Cr(VI); (f) Cr 2p high resolution XPS spectra of the Cr(VI)-adsorbed NMGO.

Fractional factorial design.

Source	Sum of squares	df	Mean Square	F Value	p-Value
Model	30460.46	9	3384.50	52.66	< 0.0001
A	21234.32	1	21234.32	330.39	< 0.0001
B	773.95	1	773.95	12.04	0.0133
C	2412.28	1	2412.28	37.53	0.0009
D	9.06	1	9.06	0.14	0.7202
E	917.18	1	917.18	14.27	0.0092
AC	3235.90	1	3235.90	50.35	0.0004
BD	402.00	1	402.00	6.25	0.0465
BE	473.72	1	473.72	7.37	0.0349
CD	1002.04	1	1002.04	15.59	0.0076
Residual	385.62	6	64.27		
Cor Total	30846.07	15			
R^2	0.9875				
Adj. R^2	0.9687				
Pred. R^2	0.9111				
Adeq. Precision	20.821				
Std. Dev.	8.02				

Table S2. ANOVA analysis of the selected factors on the adsorption efficiency of NMGO for Cu(II) in FFD.

Source	Sum of squares	df	Mean Square	F Value	p-Value
Model	6015.55	6	1002.59	152.95	< 0.0001
A	3361.59	1	3361.59	512.82	< 0.0001
B	1035.13	1	1035.13	157.91	< 0.0001
C	1242.56	1	1242.56	189.56	< 0.0001
AB	204.26	1	204.26	31.16	0.0003
AC	49.54	1	49.54	7.56	0.0225
BC	122.46	1	122.46	18.68	0.0019
Residual	59.00	9	6.56		
Cor Total	6074.54	15			
R^2	0.9903				
Adj. R^2	0.9838				
Pred. R^2	0.9693				
Adeq. Precision	37.025				
Std. Dev.	2.56				

Table S3. ANOVA analysis of the selected factors on the adsorption efficiency of NMGO for Cr(VI) in FFD.

Run number	Coded values of independent variables				
	A	B	C	D	E
1	−	+	+	−	+
2	+	−	+	−	+
3	−	+	+	+	−
4	−	−	+	−	−
5	+	−	+	+	−
6	−	−	+	+	+
7	+	+	−	−	+
8	+	+	+	+	+
9	−	+	−	+	+
10	+	+	−	+	−
11	−	−	−	+	−
12	−	−	−	−	+
13	−	+	−	−	−
14	+	−	−	+	+
15	+	+	+	−	−
16	+	−	−	−	−

Table S4. Experimental design matrix of the 2^{5-1} FFD with resolution V.

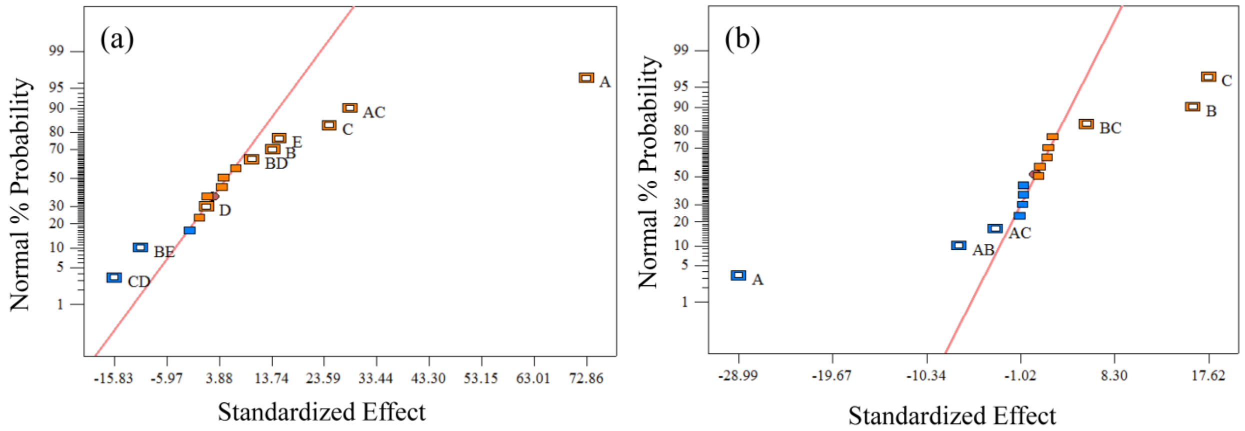

Figure S8. Normal probability plot of the effects of the factors and their interactions on the adsorption of (a) Cu(II) and (b) Cr(VI).

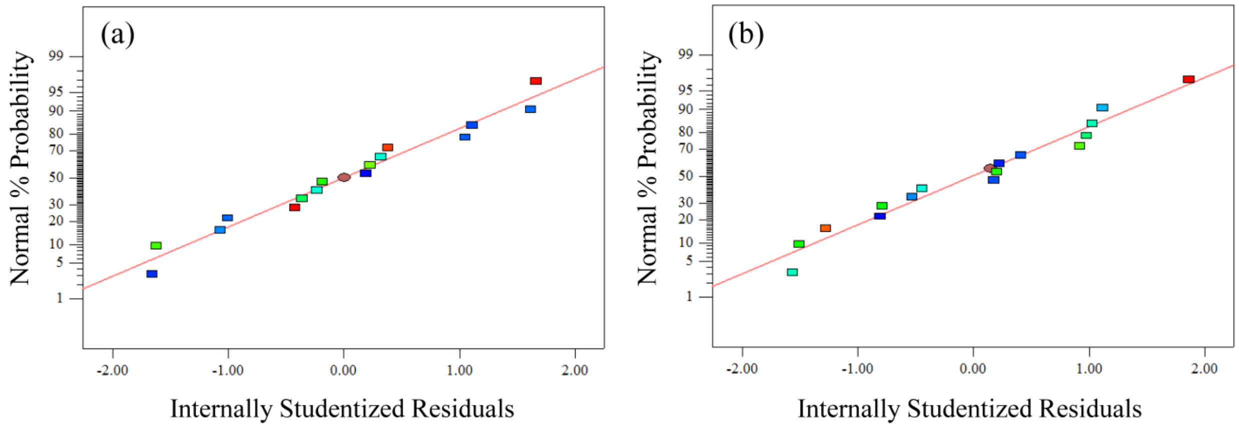

Figure S9. Plot of normal probability of residuals: (a) Cu(II) adsorption and (b) Cr(VI) adsorption.

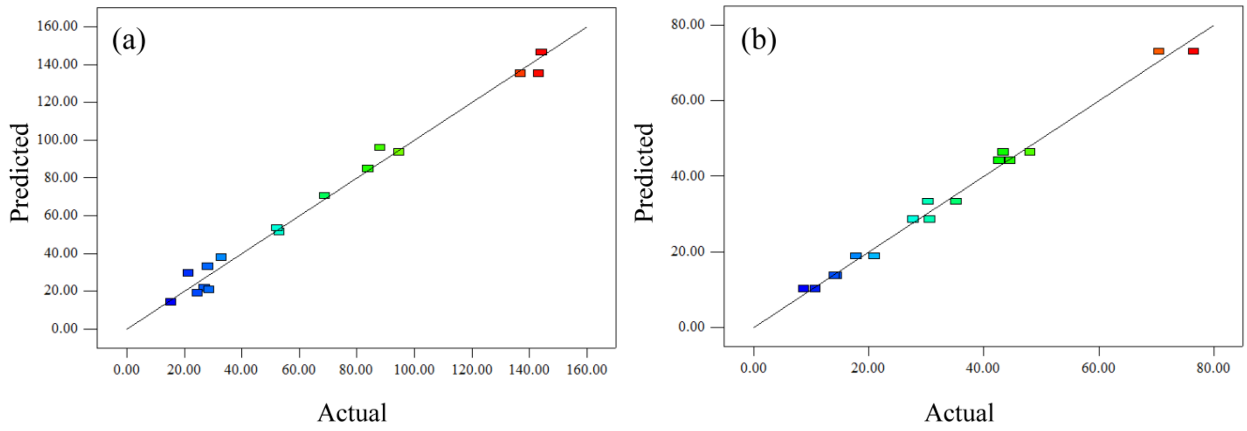

Figure S10. Comparison of predicted and experimental adsorption capacities of NMGO for (a) Cu(II) and (b) Cr(VI).

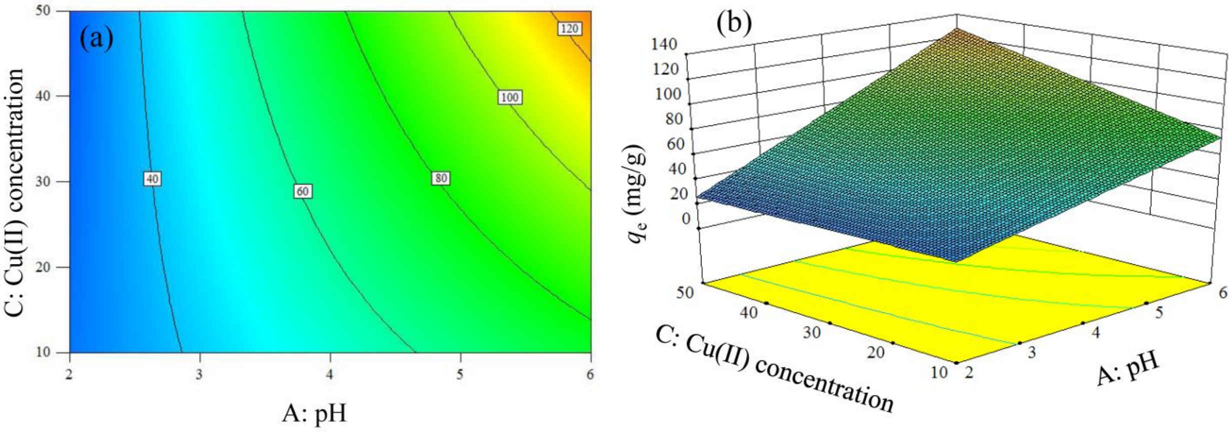

Figure S11. (a) 2D contour curves and (b) 3D surface response plot for AC interaction in the Cu(II) adsorption: temperature = 30 ℃, $CaCl_2$ concentration = 55 mmol/L, HA concentration = 5.5 mg/L.

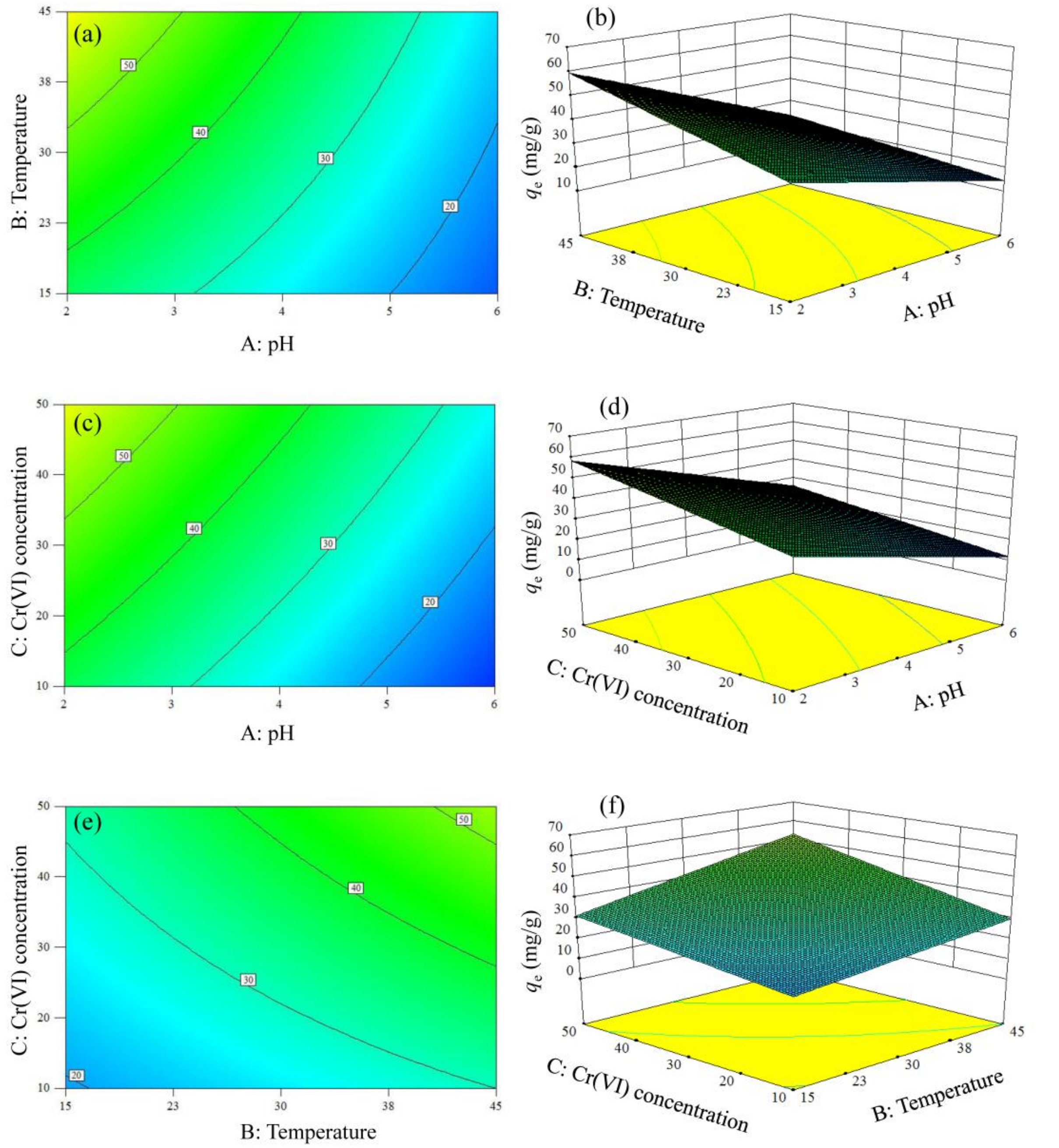

Figure S12. (a) 2D contour curves and (b) 3D surface response plot for AB interaction in the Cr(VI) adsorption: initial concentration of copper = 30 mg/L, $CaCl_2$ concentration = 55 mmol/L, HA concentration = 5.5 mg/L; **(c) 2D contour curves and (d) 3D surface response plot for AC interaction:** temperature = 30 ℃, $CaCl_2$ concentration = 55 mmol/L, HA concentration = 5.5 mg/L; **(e) 2D contour curves and (f) 3D surface response plot for BC interaction:** pH = 4, $CaCl_2$ concentration = 55 mmol/L, HA concentration = 5.5 mg/L.

References

1. Xing, H. T., *et al.* NH_2-rich polymer/graphene oxide use as a novel adsorbent for removal of Cu(II) from aqueous solution. *Chem. Eng. J.* **263**, 280-289(2015).
2. Zhao, G. X., Li, J. X., Ren, X. M., Chen, C. L. & Wang, X. K. Few-layered graphene oxide nanosheets as superior sorbents for heavy metal ion pollution management. *Environ. Sci. Technol.* **45**, 10454-10462(2011).
3. Ma, H. L., *et al.* Chemical reduction and removal of Cr(VI) from acidic aqueous solution by ethylenediamine-reduced graphene oxide. *J. Mater. Chem.* **22**, 5914-5916(2012).
4. Badruddoza, A. Z., Shawon, Z. B., Tay, W. J., Hidajat, K. & Uddin, M. S. Fe_3O_4/cyclodextrin polymer nanocomposites for selective heavy metals removal from industrial wastewater. *Carbohydr. Polym.* **91**, 322-332(2013).
5. Wang, J.-s., Peng, R.-t., Yang, J.-h., Liu, Y.-c. & Hu, X.-j. Preparation of ethylenediamine-modified magnetic chitosan complex for adsorption of uranyl ions. *Carbohydr. Polym.* **84**, 1169-1175(2011).
6. Bai, L. Z., *et al.* Inductive heating property of graphene oxide-Fe_3O_4 nanoparticles hybrid in an AC magnetic field for localized hyperthermia. *Mater. Lett.* **68**, 399-401(2012).
7. Hu, X.-j., *et al.* Effect of aniline on cadmium adsorption by sulfanilic acid-grafted magnetic graphene oxide sheets. *J. Colloid Interface Sci.* **426**, 213-220(2014).
8. Hu, X.-j., *et al.* Effects of inorganic electrolyte anions on enrichment of Cu(II) ions with aminated Fe_3O_4/graphene oxide: Cu(II) speciation prediction and surface charge measurement. *Chemosphere* **127**, 35-41(2015).
9. Zhou, L., *et al.* Investigation of the adsorption-reduction mechanisms of hexavalent chromium by ramie biochars of different pyrolytic temperatures. *Bioresour. Technol.* **218**, 351-359(2016).

10. Chen, T., *et al.* Insight into highly efficient coremoval of copper and p-nitrophenol by a newly synthesized polyamine chelating resin from aqueous media: competition and enhancement effect upon site recognition. *Environ. Sci. Technol.* **47**, 13652-13660(2013).
11. Liu, S., Wang, H., Chai, L. & Li, M. Effects of single- and multi-organic acid ligands on adsorption of copper by Fe_3O_4/graphene oxide-supported DCTA. *J. Colloid Interface Sci.* **478**, 288-295(2016).
12. Huang, B., *et al.* Synthesis of graphene oxide decorated with core@double-shell nanoparticles and application for Cr(VI) removal. *RSC Adv.* **5**, 106339-106349(2015).
13. Siva, T., Kamaraj, K., Karpakam, V. & Sathiyanarayanan, S. Soft template synthesis of poly(o-phenylenediamine) nanotubes and its application in self healing coatings. *Prog. Org. Coat.* **76**, 581-588(2013).
14. Gong, J., Liu, T., Wang, X., Hu, X. & Zhang, L. Efficient removal of heavy metal ions from aqueous systems with the assembly of anisotropic layered double hydroxide nanocrystals@carbon nanosphere. *Environ. Sci. Technol.* **45**, 6181-6187(2011).
15. Wu, J., *et al.* Chemically controlled growth of porous CeO_2 nanotubes for Cr(VI) photoreduction. *Appl. Catal. B: Environ.* **174-175**, 435-444(2015).
